# Fully Automated Diagnosis of Acute Myocardial Infarction Using Electrocardiograms and Multimodal Deep Learning

**DOI:** 10.1016/j.jacadv.2025.102011

**Published:** 2025-07-17

**Authors:** Lukas Hilgendorf, Petur Petursson, Erik Andersson, Aidin Rawshani, Deepak L. Bhatt, Truls Råmunddal, Vibha Gupta, Kristofer Skoglund, Elmir Omerovic, Helen Sjöland, Amar Taha, David Kim, Peter Lundgren, Araz Rawshani

**Affiliations:** a Institute of Medicine, Department of Molecular and Clinical Medicine, Sahlgrenska Academy, University of Gothenburg, Gothenburg, Sweden; b Wallenberg Centre for Molecular and Translational Research (WCMTM), Sahlgrenska Academy, University of Gothenburg, Gothenburg, Sweden; c Department of Cardiology, Sahlgrenska University Hospital, Gothenburg, Sweden; d Mount Sinai Fuster Heart Hospital, Icahn School of Medicine at Mount Sinai, New York, New York, USA; e Department of Emergency Medicine, Stanford University, Stanford, California, USA; f Centre for Digital Health, Sahlgrenska University Hospital, Gothenburg, Sweden

## Abstract

**Background:**

Rapid detection of acute myocardial infarction (AMI) reduces morbidity and mortality. Deep learning may enhance automated electrocardiogram (ECG) interpretation.

**Objectives:**

The purpose of the study was to develop and validate a deep learning model for AMI detection using ECG data, demographics, and symptoms.

**Methods:**

This retrospective cohort study used ECG data from 2 centers in Västra Götaland County, Sweden (January 2015-June 2023), for model training and validation, with a third center for external testing. Patients with chest pain or dyspnea who received a prehospital or in-hospital ECG were included. A residual convolutional neural network was trained on ECG features, age, sex, and symptoms to predict AMI, defined by International Classification of Diseases codes at discharge. Performance was assessed using area under the receiver operating characteristic, sensitivity, and specificity.

**Results:**

The study included 104,507 individuals (208,366 ECGs), with 8.17% in the training set and 8.59% in the external set diagnosed with AMI. The model achieved AUROCs of 0.8221 ± 0.0101 (internal validation ± 95% CI) and 0.8314 ± 0.0085 (external validation). Performance was consistent across sex but slightly lower for ambulance-arriving patients (area under the receiver operating characteristic: 0.8081 ± 0.0095). Saliency maps highlighted focus on ST segments and T waves.

**Conclusions:**

The deep learning model demonstrated strong AMI detection across diverse patient groups. A randomized trial is needed to compare its performance with emergency physicians.

Acute myocardial infarction (AMI) remains a leading cause of mortality worldwide. The majority of these deaths are caused by ventricular arrhythmias occurring during the first few hours, emphasizing the need for timely and accurate diagnosis.^1^ The electrocardiogram (ECG) is fundamental for diagnosis, risk stratification, and management, particularly in the early phase prior to when troponin results are available. Studies demonstrate that a physician’s ability to correctly diagnose AMI, as measured by the area under the curve (AUC) of the receiver operating characteristic (ROC), is between 0.72 and 0.80.^2^, ^3^, ^4^ This underscores the need for new methods for evaluating the ECG.

Modern electrocardiographs often incorporate digital ECG interpretation algorithms, which predominantly rely on predefined features, fixed thresholds, and rule-based logic. More recently, advancements in deep learning have led to the development of sophisticated models for ECG analysis, both in arrhythmia classification and AMI detection. A convolutional neural network is a type of neural network that has demonstrated exceptional ability in pattern recognition and has therefore been used extensively for ECG applications.^5^, ^6^, ^7^

To date, deep learning models for detecting AMI have been predominantly unimodal, relying solely on the ECG signal. This results in the loss of critical predictive information inherent in factors such as age, sex, and symptoms—features that are universally available for all patients with suspected AMI and relevant to the diagnosis.^8^, ^9^, ^10^, ^11^, ^12^ Incorporating these factors may allow for AI models to make more accurate predictions.

To the best of our knowledge, we used the largest cohort to date to train a multimodal neural network, taking into account the ECG as well as patient age, sex, and symptoms, to create a fully automated algorithm for patients with suspected AMI.

## Methods

### Study population

In this retrospective cohort study, we included all patients with chest pain or dyspnea who received a 12-lead ECG within 6 hours of admission to the emergency room (ER) or a prehospital ECG in the 2 hours prior to ER admission, at 3 centers (Sahlgrenska Universitetsjukhus, Norra Älvsborgs Länssjukhus, and Skaraborgs Sjukhus) in the Swedish county of Västra Götaland for chest pain or dyspnea between January 1, 2015 and June 30, 2023. If both a prehospital and an in-hospital ECG were available in the given time frame, both were included. If multiple in-hospital or multiple prehospital ECGs were available, the one closest to ER admission was selected for each category to capture the ECG taken in triage or the last available prehospital ECG. This was done as, in a prehospital setting, multiple ECG readings can indicate quality issues in the first ECG taken; therefore, the last reading was selected.

### ECG preprocessing

ECGs were received in XML format encoded in base 64 and a corresponding sampling frequency; they were converted to base 10 values in millivolts and WFDB format.^13^

The recording frequency (Hertz [Hz]) of the ECGs varied across hospitals and ambulance. Therefore, we performed downsampling to 250 Hz, using cubic spline interpolation.^14^, ^15^, ^16^ ECGs were then normalized using z-score normalization to avoid the problem of amplitude scaling, as done by previous studies.^17^, ^18^, ^19^ This process involves subtracting the signal mean and dividing by the SD to achieve a new signal mean of 0 and an SD of 1. We chose to further reduce amplitude variability and divide by twice the SD to achieve a new SD of 0.5, as described in previous research.^20^

### Data labels and multimodality

Labels for individual patients were obtained using final discharge codes (International Classification of Diseases-10 codes) from the ER or hospital stay. A patient belonged to the positive class (ie, had experienced an AMI) if ICD-10 code I21 was recorded.^21^

The multimodality of the model implied including age, sex, and main symptom. Age and sex were obtained using the Swedish personal identification number; age was recorded as an integer, while sex was encoded in a binary variable. Two major symptoms were included in the study, chest pain, and dyspnea. Both symptoms were encoded as separate binary variables, based on ICD-10 codes in the ER, beginning with R07 (excluding R07.0) for chest pain and R060 for dyspnea.^22^ A patient could have both chest pain and dyspnea. The inclusion criteria of chest pain and dyspnea were taken from the triage system; however, these were not included in the data from the hospitals; therefore, ICD codes were used to generate symptom labels.

### Model development

**Figure 1:**
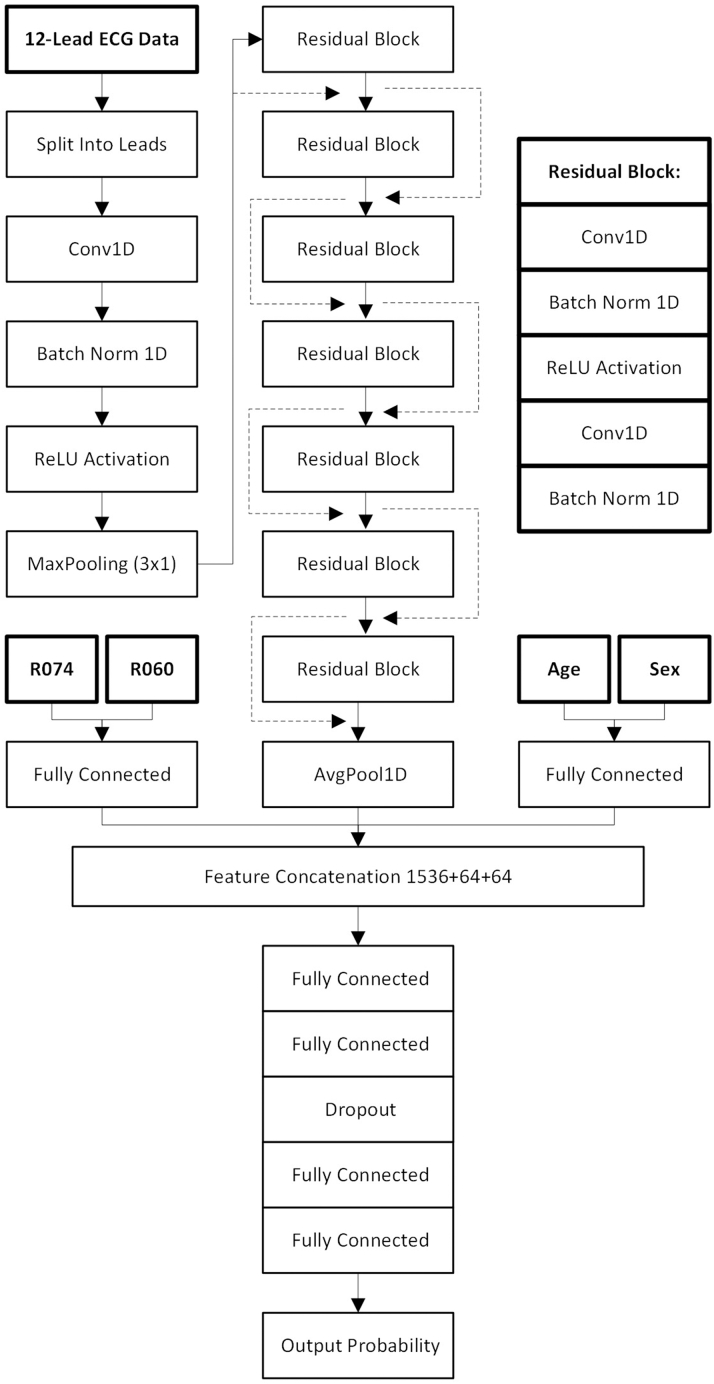
Final Model Architecture Architecture of the final model: R074 represents a binary variable for chest pain, while R060 represents a binary variable for dyspnea. Each residual block in the algorithm is constructed according to the substructure to the right in the figure, with the dotted lines representing the skip connections. ECG = electrocardiogram.

The model architecture was developed based on a residual convolutional neural network structure, widely used in ECG analysis.^23^, ^24^, ^25^ This extracted features from the ECG. Fully connected layers extracted features from the binary variables sex, chest pain, dyspnea, and the integer variable age. These features then go through a fully connected neural network for classification. The full model architecture can be seen in Figure 1. The architecture was developed in an iterative approach to optimize for depth and layer types. The model was trained with an ADAM optimizer, and learning rates between 0.001 and 0.00001 were evaluated in steps decreasing to one-tenth of the prior attempt. Dropout rates were tested in increments of 0.1 between 0.0 and 0.5.

The data from 2 hospitals (Sahlgrenska Universitetsjukhus and Skaraborgs Sjukhus) were included as training and internal validation data, while data from the third hospital (Norra Älvsborgs Länssjukhus) were used for external validation. The internal data were split into an 80-10-10, training, testing, and validation data. The split was performed based on the personal identification number to avoid data leakage, as we expected the prehospital and in-hospital ECGs for 1 patient to show autocorrelation.^26^

To prevent overfitting, early stopping was implemented, with a minimum number of 10 epochs and a stopping function activating if validation loss has not improved over the past 10 epochs.^27^

All of the models were created using the PyTorch^28^ software package on one NVIDIA RTX 4090.

### Model evaluation

For the purpose of evaluation, each model was trained 10 times, each time on a different split of the same inputs. Uniqueness of the splits was ensured by specifying 10 different randomly generated seeds, excluding the seeds that had already been run. This allows for a mean with 95% CI to be calculated for all metrics to be computed across the models and ensures that the model performs independent of the input data split.

The model was evaluated primarily utilizing the AUC of the ROC. This was done for both the internal testing and external validation data to compare the performance across the data sets. All other analysis was performed solely on the external validation data. This further validation included test characteristics (area under the precision-recall curve [AUPRC], negative predictive value, positive predictive value, F1-score, sensitivity, and specificity) at the operating point maximizing Youden J statistic to optimize for both sensitivity and false positive rate simultaneously.^29^ The F1-scores were computed as macro F1-scores to give equal weight to the positive and negative classes despite an expected class imbalance.^30^ The model was also tested on specific subgroups of the external validation data, namely sex-specific and in-hospital vs prehospital ECGs, to determine if the model is applicable to all major groups included in the study population.

To determine the benefit of multimodality, the models were trained 5 times with only access to ECG data, 5 times without access to demographic data, and 5 times without access to symptom data. The first 5 random data splits from the main model were used for training; the evaluation was then performed on the same external validation data as the main model.

### Qualitative evaluation

Saliency maps were utilized to determine which segments of the ECG are important for the model’s prediction. These were computed over 10 randomly selected samples of each class from the external validation set. From these, the central beat was extracted and plotted beside each other to create an overview of the models’ focus for different classes. Saliency maps were not averaged nor overlaid so as not to drown out possible outliers but to allow for an examination of all different ECGs individually.

## Results

**Figure 2:**
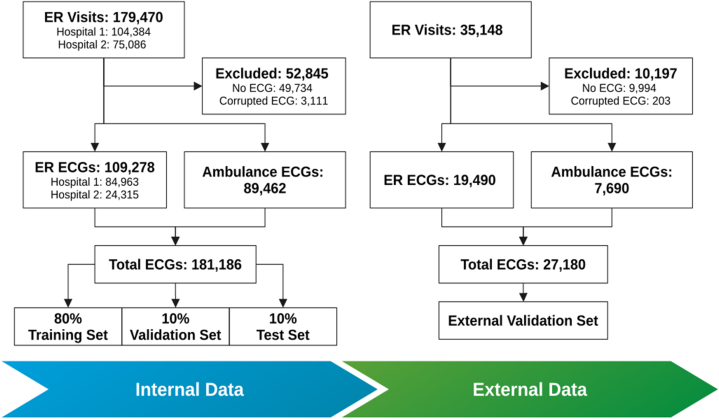
Data Set Curation Description of the process of data set creation for both the internal and external sets, describing inclusion and exclusion. ECG = electrocardiogram; ER = emergency room.

The study included 85,324 individuals, contributing 181,186 ECGs for training and internal validation, and 19,183 individuals contributing 27,180 ECGs for external validation. A total of 6,968 individuals (8.17%) experienced an AMI in the training set, and 1,647 individuals (8.59%) in the external validation set. The pathway to inclusion and exclusion is presented in Figure 2. The characteristics of the features used by the model were comparable across the sets (described in Table 1).

**Table 1:** Description of the Data Sets

	Internal Hospitals Training, Validation, and Test Data		External Hospital Validation Data	
	AMI (n = 11,802, 6.51%)	No-AMI (n = 169,384, 93.49%)	AMI (n = 2,208, 8.12%)	No-AMI (n = 24,972, 91.88%)
Age (y)	72.78 ± 13.02	64.64 ± 19.15	74.30 ± 13.19	62.07 ± 19.19
Unique patients	6,968	78,356	1,647	17,536
Chest pain	6,042 (51.19%)	90,800 (53.61%)	1,326 (60.05%)	17,542 (70.25%)
Dyspnea	1,033 (8.75%)	44,449 (26.24%)	289 (13.09%)	7,184 (28.77%)
Prehospital ECGs	6,770 (57.36%)	82,692 (48.82%)	774 (35.05%)	6,916 (27.70%)
Male	7,771 (65.84%)	85,521 (50.49%)	1,395 (63.18%)	13,021 (52.14%)

Values are mean ± SD, n, or n (%).

AMI = acute myocardial infarction; ECG = electrocardiogram.

The final model was trained with a learning rate of 0.001 and a dropout rate of 0.0, as this yielded the highest internal testing results. The dropout rate of 0.0 implies that the dropout layer was inactive in the final model; it was included in the final architecture, however, as the difference was slim and previous iterations showed significant improvement when applying a dropout.

The mean training and validation loss over epochs with their respective 95% CI can be seen in Supplemental Figure 1, together with a representation of after which epoch runs were stopped. A slight divergence in loss seems to appear after training for 24 epochs; however, for most runs, the model stopped before reaching this point.

**Figure 3:**
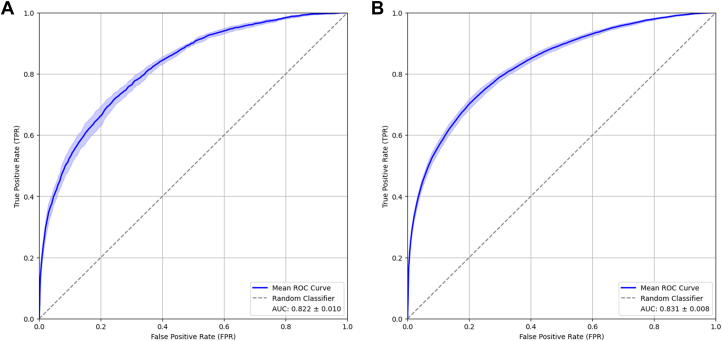
Receiver Operating Characteristic Curves for Internal and External Testing Data ROC curve over 10 models of the same architecture (mean ± 95% CI), each trained on a unique split of the data into testing and training data, (A) for the internal testing set and (B) for the external validation set for the model predicting AMI from 12-lead ECG obtained from patients presenting to an emergency room with chest pain or dyspnea. The AUC of 0.822 ± 0.010 (mean ± 95% CI) indicates adequate prognostic power, as the AUC under the line of identity (0.5) represents full randomness of prediction, that is, zero predictive power of the model. AMI = acute myocardial infarction; AUC = area under the curve; ECG = electrocardiogram; ROC = receiver operating characteristic.

**Figure 4:**
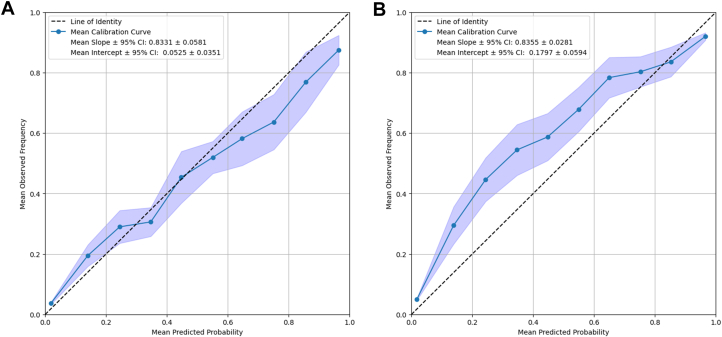
Calibration Curves for Internal and External Testing Data Calibration curve over 10 models of the same architecture (mean ± 95% CI), each trained on a unique split of the data into testing and training data, for (A) the internal testing set and (B) the external validation set for the final neural network predicting AMI from 12-lead ECG obtained from patients presenting to an emergency room with chest pain or dyspnea. Abbreviations as in Figure 3.

The ROC curve for internal testing can be seen in Figure 3A, yielded an average AUC of 0.8221 ± 0.0101. The external validation set yielded a mean ROC represented in Figure 3B, with an AUC of 0.8314 ± 0.0085. The internal testing calibration curve, Figure 4A, yielded a mean slope of 0.8331 ± 0.0581 and a mean intercept of 0.0525 ± 0.0351. The external validation set also yielded a calibration curve, Figure 4B, and a mean slope of 0.8355 ± 0.0281 and mean intercept of 0.1797 ± 0.0594. This indicates the model is well calibrated on the internal data while underpredicting on the external data.

An analysis of the model without non-ECG features yielded an AUC of 0.7738 ± 0.0158, the inclusion of only sex and age yielded an AUC of 0.8122 ± 0.0132; and the inclusion of only symptoms resulted in an AUC of 0.7912 ± 0.0232, averaged over 5 training runs on different training splits of the same data and evaluated on the same external validation set as the final model.

The total and subgroup analyses for the external validation set are presented in Table 2. It presents a consistent picture across subgroups with low variance between the analyzed groups. The only subgroup with a clearly decreased area under the receiver operating characteristic was the ambulance subset, while the lowest sensitivity but the highest specificity was achieved in the female-only subset. The subsets of prehospital ECGs and ER ECGs display performance on a level where each label is used to only define 1 ECG, as 1 visit never leads to multiple ER ECGs or multiple prehospital ECGs being included.

**Table 2:** Subgroup Analyses Within the External Validation Data

	AUROC	AUPRC∗	Sensitivity	Specificity	F1-Score	NPV	PLR	NLR
Whole data								
Overall	0.8314 ± 0.0085	0.4581 ± 0.0121	0.7255 ± 0.0165	0.7804 ± 0.1792	0.6066 ± 0.0122	0.9696 ± 0.0013	3.3470 ± 0.2312	0.3514 ± 0.0166
Subset								
Male	0.8317 ± 0.0093	0.4850 ± 0.0151	0.7242 ± 0.0281	0.7762 ± 0.0308	0.6304 ± 0.0143	0.9633 ± 0.0025	3.3383 ± 0.3324	0.3535 ± 0.0250
Female	0.8242 ± 0.0098	0.4167 ± 0.0151	0.6813 ± 0.0173	0.8161 ± 0.0111	0.5813 ± 0.0146	0.9740 ± 0.0012	3.7291 ± 0.1898	0.3903 ± 0.0012
Only ER ECGs	0.8379 ± 0.0085	0.4494 ± 0.0155	0.7259 ± 0.0120	0.7907 ± 0.0163	0.6018 ± 0.0114	0.9732 ± 0.0009	3.5099 ± 0.2337	0.3465 ± 0.0121
Only prehospital ECGs	0.8081 ± 0.0095	0.4640 ± 0.0129	0.7081 ± 0.0362	0.7675 ± 0.0349	0.6359 ± 0.0109	0.9597 ± 0.0032	3.1583 ± 0.3233	0.3778 ± 0.0311

All values describe the mean over 10 runs ±95% CI.

AUPRC = area under the precision-recall curve; AUROC = area under the receiver operating characteristic; ECG = electrocardiogram; ER = emergency room; NLR = negative likelihood ratio; NPV = negative predictive value; PLR = positive likelihood ratio.

∗ Random classifier on this data set would achieve an AUPRC of 0.0651 (prevalence of positive class).

Care needs to be taken when evaluating the AUPRC, as it needs to be viewed in light of the prevalence of the positive class in the training population, in this case 6.51% (Table 1), meaning a random classifier would achieve an AUPRC of 0.0651. It is also to be noted that AUPRC heavily favors the majority subgroup.^31^ For a plot of the precision-recall curve, see Supplemental Figure 2.

**Figure 5:**
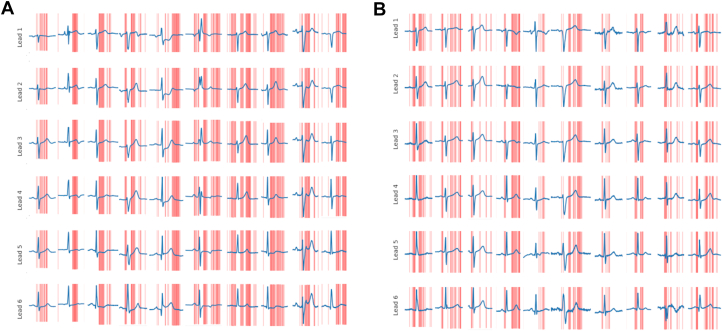
Saliency Maps Saliency maps, calculated from 10 randomly selected ECGs from the external validation set—10 beats from the middle of separate ECGs side by side for comparison of extracted features in (A) the positive class and (B) the negative class. Abbreviation as in Figure 3.

Figure 5, shows the saliency maps of the central beats of 10 ECGs of the positive class (panel A) and the negative class (panel B). The ECGs were randomly selected from the external validation set based on their labels; 2 were removed and replaced from the non-AMI group due to strong artifacts in the ECG making human interpretation more difficult. The excluded saliency maps can be found in the Supplemental Figure 3. The final maps show a clear focus on the ST-segment and T-wave of the ECG as well as the PR interval. This is in line with the guidelines set forth for human interpretation of ECG, which focuses mainly on the ST-segment and the T-wave.

**Central Illustration:**
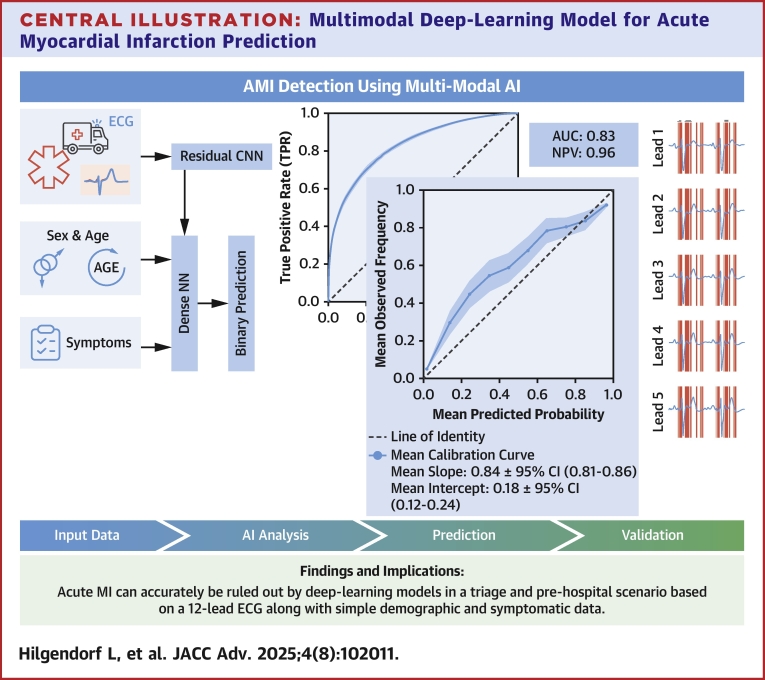
Multimodal Deep-Learning Model for Acute Myocardial Infarction Prediction AI = artificial intelligence; CNN = convolutional neural network; NN = neural network; NPV = negative predictive value; other abbreviations as in Figure 3.

## Discussion

We developed and validated a deep learning model capable of exceeding the previously reported performance of physicians with regard to diagnosing AMI. Our model was trained on a diverse population of both males and females with chest pain and dyspnea, using both in- and out-of-hospital (ambulance) ECGs, thus reflecting the patient population in an ER. The model presented in this study has the advantage of a large underlying data set, with a significant external validation set. This allows for the interpretation of the results as robust and applicable to the real-world setting. Indeed, given the model performance, this model could be deployed to the clinical setting. The model is well calibrated, with performance required for clinical use (Central Illustration).

The presented model also benefits from taking into consideration multiple relevant modalities in conjunction with the ECG. This puts it in the minority, as most published models, as only 20% of ECG algorithms employ additional data streams.^32^ The addition of these variables also markedly improved performance, with the age and sex variables being the largest contributors. These are also the easiest to implement in clinical practice, as other variables can be a lot more time-consuming to record and input, whereas age and sex can in many cases be extracted from patient ID numbers or an electronic charting system where they almost always are recorded in a standardized fashion.

It is essential to recognize that this model predicts the final diagnosis of AMI regardless of the underlying mechanism. This means it encompasses myocardial infarctions caused by total occlusions (typically ST-elevation myocardial infarction), partial occlusions (typically non-ST-elevation myocardial infarction), and other etiologies of AMI. The diversity in outcomes highlights the model’s robust predictive performance. Hence, this model predicts a final diagnosis of AMI in any patient presenting to the ER with chest pain or dyspnea, irrespective of subtype of infarction.

The saliency maps provide insight into the models’ extracted features and indicate segments corresponding to guidelines for AMI detection, with a focus on the ST-segment and T-wave. This lends confidence to the interpretation that the model produces. The model’s focus on the PR interval suggests that the model correctly captures the relevance of the isoelectric (reference) line for measuring ST-T deviations. An analysis using multiple overlayed maps was avoided to enable a case-by-case comparison for a realistic individual ECG model interpretation. Our goal is, if applied clinically, to enable an overlay for each analyzed ECG. This would allow the interpreter of the result to check if the model is behaving as expected if the predicted result or ECG quality is cause for concern.

The difference in AUC between the ambulance and ER subsets may reflect that these are 2 different patient populations, with those arriving by ambulance exhibiting more comorbidities, greater prevalence of cardiovascular disease, and poorer outcomes, as previously demonstrated.^33^ This underscores the importance of having even larger training data volumes, which we are planning for in a subsequent analysis. It could also stem from the fact that most ER ECGs were sampled at 500 Hz, whereas most ambulance ECGs were sampled at 400 Hz. Thus, during downsampling, more of the ambulance ECGs underwent interpolation as the 500 Hz ECGs were resampled to only include every second data point. Yet another explanation could be that Mason-Likar electrode placement is commonly used in the ambulance, meaning that electrodes are relocated to the torso instead of extremities. This has been shown to affect the ST-T segment and QRS complex.^34^^,^^35^ Thus, ECGs in the ambulance and the ER may represent slightly different patient populations, different sampling rates and method for signal recording, which may have induced the discrepancies in performance.

Models with similar purposes have been presented in the past.^3^ Notably, the ECG-SMART system reached external validation AUC between 0.85 and 0.90. This model was, however, trained on a rather selected subset of patients, namely those presenting in the ambulance who did not have STEMI and who had a confirmed coronary artery occlusion on coronary angiography. Hence, the ECG-SMART system was trained on a subset of patients who is not possible to identify at the point of first medical contact. Because these patients exhibit occlusions, they should present with more pronounced ST-T changes on their ECG.^36^ Another deep learning model has achieved an AUC of 0.975 for anterior-STEMI detection; however, this was in a highly selected database on which all physicians and algorithms that they compared their model to performed markedly better than in a hypothetical real world where the class sizes were adapted to better represent those of the real world, resulting in an AUC of 0.586 for the model, indicating the importance of training on data and class sizes resembling the real world.^37^ This is also why the class imbalance in the data set was not corrected for in training and testing, but rather to view the results in light of this after the fact, to ensure that any results would be as close to a real-world scenario as possible. Their model, however, saw improvement when combined with information from troponin testing, yielding more accurate results than model prediction or troponin testing alone. This promises that the combination of highly accurate prediction of troponin elevation, as we have done in the past,^20^ and of AMI may yield even improved predictive power.

For future studies, this model could be further improved by enlarging the data set further, as while containing a substantial number of patients, the percentage of those presenting with AMI is low, varying between the data sets between 6% and 8%. This yields difficulty in ensuring that models with architectures as deep as the one presented here can truly make use of the deepest layers. Therefore, these models benefit from larger data sets to avoid vanishing gradients. A common method of solving this issue of small data sets today is transfer learning, where a pretrained model is adapted and fine-tuned on a specific task.^38^ In this case, however, to ensure the integrity of the model and to make it more easily understood by guaranteeing that we know exactly what data the model has been trained on and how and why these data were selected, allowing us to predict with a greater degree of certainty that it will function in a similar fashion if implemented clinically.

### Study Limitations

Our model had challenges in certain instances, as can be seen when evaluating the calibration curve for the external validation set, with a constant underprediction of risk by 10% to 20% until it rejoins the line of identity in the predicted range of 80% to 100%. This is suboptimal for clinical implementation, as this could leave patients exposed to underdiagnosis. Furthermore, while the AUC of 0.83 is robust and constant over internal and external validation data and greater than that of most clinicians, there remains room for improvement.

The very limited amount and complexity of exclusion criteria of the data set, while guaranteeing as close to real evaluation as possible, does bring along the issues associated with data containing artifacts and poor quality ECGs, while further introducing other ECG pathologies not relating to AMI, such as arrhythmias and bundle branch blocks. The choice to not prefilter the data set for artifacts and previous ECG pathologies was a conscious one, so as to not put a burden on a potential end-user to decide whether or not the model can be applied to a certain patient based on ECG morphology and prior medical history, but rather to have simple inclusion criteria based on being an adult and exhibiting the symptoms that were inclusion criteria for the study. The unfiltered nature of the data set also introduces ECGs that may have been rerecorded due to different issues such as low-voltage, base-line drift, and other artifacts, especially as the ECGs excluded for corruption were only those that could not be decoded from their storage format, not filtering out any other ECGs. These may impact measured model performance and present as false positives or negatives, as the crucial information for classification may be lost. It could, however, be argued that these will not be seen by the model in a real-world setting, as the ECGs would be rerecorded by the nursing staff, who are trained to check the quality of their recordings.

For the study, we selected to only include age, sex, and symptoms as copredictors; this list was not expanded further to include comorbidities and medications, even though this could increase model performance. Primarily to retain clinical and prehospital usability, as resources are limited and the input of extra data would take up valuable resources. Furthermore, it is difficult to collect high-quality data retrospectively and ensure that a patient was taking certain medications or was knowingly suffering from certain comorbidities at the time of triage or in the ambulance.

The data set displays a discrepancy between the expected number of patients presenting with symptoms and recorded symptoms. This stems from how the symptom labels were created, as we did not have access to the symptoms listed on triage forms, which were used to define the data set, but rather relied on the recording of symptoms as a diagnosis in the ER. This is known to be inconsistent and unreliable, as it depends on the attending physician if they choose to record symptoms as diagnoses or not. We decided to explore the addition of symptoms anyways to see if it would improve model performance, resulting in slightly increasing the AUC from 0.81 to 0.83. Therefore, we included it in the final model architecture, as we believe that there may be even greater value in this data in future studies with more accurate labels.

## Conclusions

This study presents a novel multimodal model architecture capable of excluding AMI with a high negative predictive value. We are unaware of any other model able to exclude AMI with such high precision in an equally broad patient population with suspected acute coronary syndromes. The model performs consistently across an internal and an external data set, across sexes and other tested subgroups. Next, this model needs to be evaluated in a pragmatic randomized trial.

Perspectives **COMPETENCY IN SYSTEMS-BASED PRACTICES:** The existing system for processing AMI patients involves delays due to factors such as slow identification and missed diagnoses on ECGs. To enhance efficiency, it is essential to streamline the process, enabling triage staff to promptly alert physicians about patients requiring urgent care. Implementing modern deep-learning systems can be instrumental in addressing these challenges.

**TRANSLATIONAL IMPLICATIONS:** Clinical implementation of this model necessitates several steps, including validation on prospective data and integration into existing or novel workflows. Evaluating these implementations will determine if they facilitate more rapid treatment through the utilization of such systems.

## Declaration of generative AI and AI-assisted technologies in the writing process

During the preparation of this work, the author(s) used ChatGPT in order to assist in and accelerate coding and debugging Python code for model creation; all of the generated code and suggestions have been checked for functionality and correctness by the corresponding author. After using this tool/service, the author(s) reviewed and edited the content as needed and take(s) full responsibility for the content of the publication.

## Funding support and author disclosures

This study was funded by the Swedish Society for Medicine (Svenska Läkarsällskapet, grant number SLS-1000926), the Swedish state under the agreement between the Swedish government and the county councils, the ALF-agreement (ALFGBG-998297), and the 10.13039/501100017018Wallenberg Centre for Molecular and Translational Medicine (WCMTM). Dr Bhatt discloses the following relationships: Advisory board: Angiowave, Bayer, Boehringer Ingelheim, CellProthera, Cereno Scientific, E-Star Biotech, High Enroll, Janssen, Level Ex, McKinsey, Medscape Cardiology, Merck, NirvaMed, Novo Nordisk, Stasys; Tourmaline Bio; Board of Directors: American Heart Association New York City, Angiowave (stock options), Bristol Myers Squibb (stock), DRS.LINQ (stock options), High Enroll (stock); Consultant: Broadview Ventures, Corcept Therapeutics, GlaxoSmithKline, Hims, SFJ, Summa Therapeutics, Youngene; Data Monitoring Committees: Acesion Pharma, Assistance Publique-Hôpitaux de Paris, Baim Institute for Clinical Research (formerly Harvard Clinical Research Institute, for the PORTICO trial, funded by St. Jude Medical, now Abbott), Boston Scientific (Chair, PEITHO trial), Cleveland Clinic, Contego Medical (Chair, PERFORMANCE 2), Duke Clinical Research Institute, Mayo Clinic, Mount Sinai School of Medicine (for the ENVISAGE trial, funded by Daiichi Sankyo; for the ABILITY-DM trial, funded by Concept Medical; for ALLAY-HF, funded by Alleviant Medical), Novartis, Population Health Research Institute; Rutgers University (for the NIH-funded MINT Trial); Honoraria: American College of Cardiology (Senior Associate Editor, Clinical Trials and News, ACC.org; Chair, ACC Accreditation Oversight Committee), Arnold and Porter law firm (work related to Sanofi/Bristol-Myers Squibb clopidogrel litigation), Baim Institute for Clinical Research (formerly Harvard Clinical Research Institute; AEGIS-II executive committee funded by CSL Behring), Belvoir Publications (Editor in Chief, Harvard Heart Letter), Canadian Medical and Surgical Knowledge Translation Research Group (clinical trial steering committees), CSL Behring (AHA lecture), Cowen and Company, Duke Clinical Research Institute (clinical trial steering committees, including for the PRONOUNCE trial, funded by Ferring Pharmaceuticals), HMP Global (Editor in Chief, Journal of Invasive Cardiology), Journal of the American College of Cardiology (Guest Editor; Associate Editor), Level Ex, Medtelligence/ReachMD (CME steering committees), MJH Life Sciences, Oakstone CME (Course Director, Comprehensive Review of Interventional Cardiology), Piper Sandler, Population Health Research Institute (for the COMPASS operations committee, publications committee, steering committee, and USA national co-leader, funded by Bayer), WebMD (CME steering committees), Wiley (steering committee); Other: Clinical Cardiology (Deputy Editor); Patent: Sotagliflozin (named on a patent for sotagliflozin assigned to Brigham and Women's Hospital who assigned to Lexicon; neither I nor Brigham and Women's Hospital receive any income from this patent); Research funding: Abbott, Acesion Pharma, Afimmune, Aker Biomarine, Alnylam, Amarin, Amgen, AstraZeneca, Bayer, Beren, Boehringer Ingelheim, Boston Scientific, Bristol-Myers Squibb, Cardax, CellProthera, Cereno Scientific, Chiesi, CinCor, Cleerly, CSL Behring, Faraday Pharmaceuticals, Ferring Pharmaceuticals, Fractyl, Garmin, HLS Therapeutics, Idorsia, Ironwood, Ischemix, Janssen, Javelin, Lexicon, Lilly, Medtronic, Merck, Moderna, MyoKardia, NirvaMed, Novartis, Novo Nordisk, Otsuka, Owkin, Pfizer, PhaseBio, PLx Pharma, Recardio, Regeneron, Reid Hoffman Foundation, Roche, Sanofi, Stasys, Synaptic, The Medicines Company, Youngene, 89Bio; Royalties: Elsevier (Editor, Braunwald’s Heart Disease); Site co-investigator: Cleerly. All other authors have reported that they have no relationships relevant to the contents of this paper to disclose.
