# Supplementary material for: Fully Automated Diagnosis of Acute Myocardial Infarction Using Electrocardiograms and Multimodal Deep Learning
Source: JACC Adv. 2025 Jul 17;4(8):102011. doi: 10.1016/j.jacadv.2025.102011 (PMC12284675; doi:10.1016/j.jacadv.2025.102011)

Supplemental Figure 1:

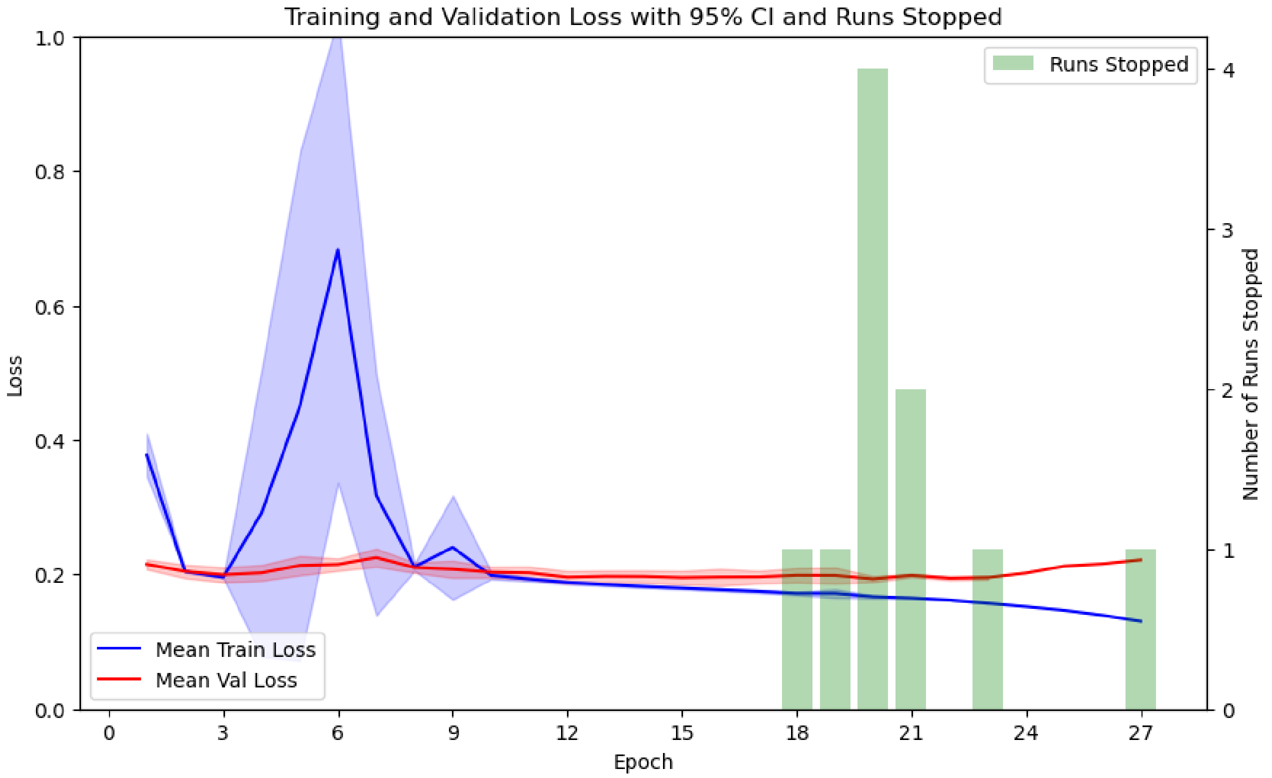

Mean training and validation loss (± 95% CI) in relation to each other and over the epochs of training, taken over all 10 different training iterations (left y-axis). Furthermore, the figure indicates after which epochs early stopping activated to prevent over fitting(right y-axis).

Supplemental Figure 2:

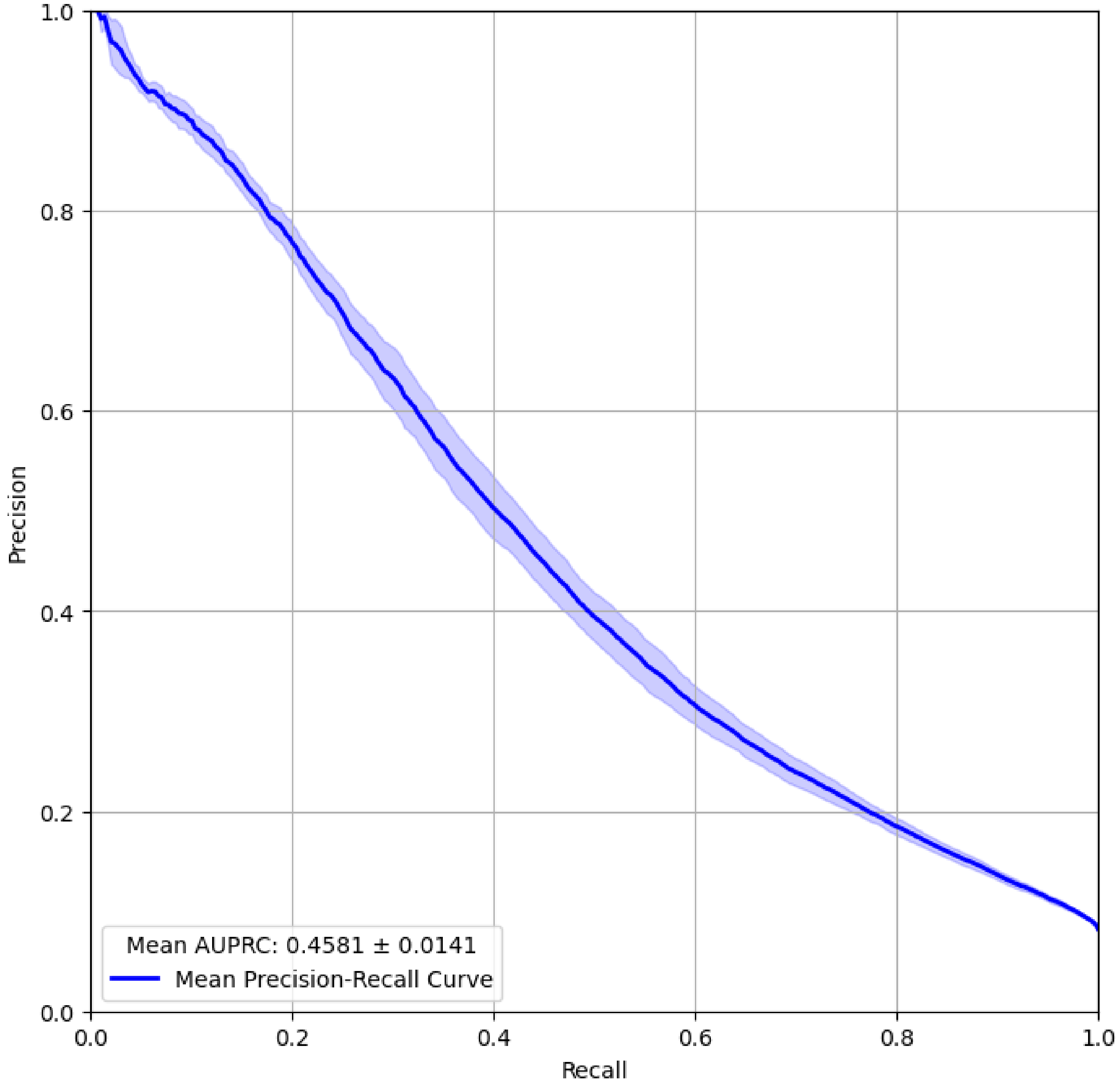

Precision Recall Curve of the over 10 models of the same architecture (mean ± 95%CI), each trained on a unique split of the data into testing and training data, for the external validation set for the model predicting AMI from 12-lead ECG obtained from patients presenting to an emergency room with chest-pain or dyspnea. The area-under the curve (AUC) of 0.458 ± 0.014 (mean ± 95% CI), indicating- adequate prognostic power, as the AUC under 0.0651 represents full randomness of prediction, i.e. zero predictive power of model (due to positive class prevalence of 6.51%).

Supplemental Figure 3:

The two saliency maps which were excluded from the non-MI group due to artifacts.

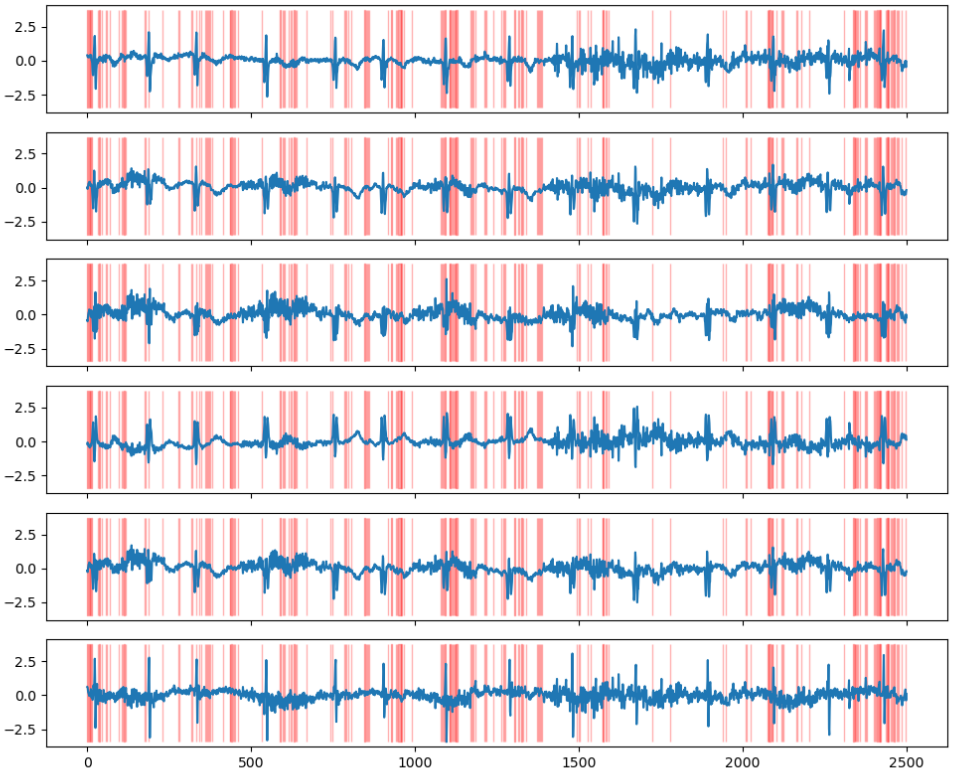

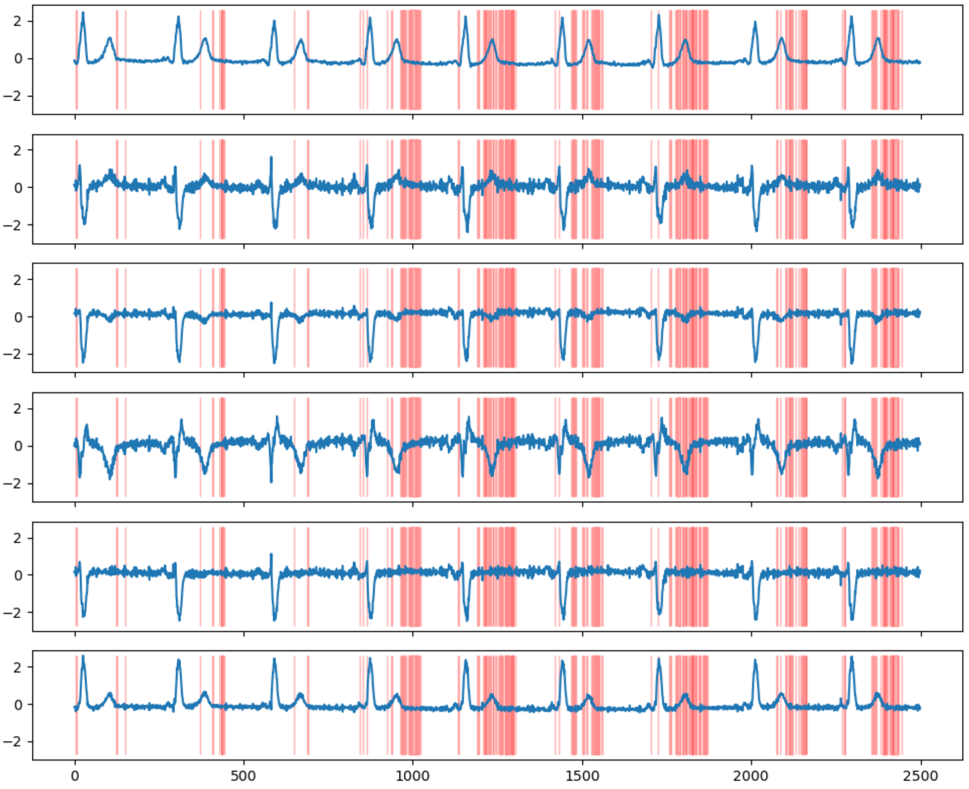

Supplement: Supplemental_Material [file mmc1.pdf]
